# A new diagnostic technique for identifying *Angiostrongylus* spp. larvae in intermediate snail species by examining the buccal cavity

**DOI:** 10.1186/s13071-024-06350-1

**Published:** 2024-07-09

**Authors:** Yong-bo Zhao, Ling Jiang, Wen Fang, Shao-rong Chen, Yu-hua Liu, Shen-hua Zhao, Peter S. Andrus, Tian-mei Li, Yun-hai Guo

**Affiliations:** 1 Institute of Schistosomiasis Prevention and Control, Dali, 671000 Yunnan Province China; 2 National Key Laboratory of Intelligent Tracking and Forecasting for Infectious Diseases, National Institute of Parasitic Diseases, Chinese Center for Disease Control and Prevention, Chinese Center for Tropical Diseases Research, Key Laboratory on Parasite and Vector Biology, Ministry of Health, WHO Centre for Tropical Diseases, National Center for International Research on Tropical Diseases, Ministry of Science and Technology, Shanghai, 200025 China; 3 School of Global Health, Chinese Center for Tropical Diseases Research, Shanghai Jiao Tong University School of Medicine, Shanghai, 200025 China

**Keywords:** *Angiostrongylus cantonensis*, Snail vectors, Lungworm detection, Gastropod-borne disease

## Abstract

**Background:**

Angiostrongyliasis is a zoonotic parasitic disease caused by the rat lungworm *Angiostrongylus cantonensis*. The intermediate hosts of *A. cantonensis* are gastropods, and snail species such as *Pomacea canaliculata* play a key role in the transmission of human angiostrongyliasis. Detecting *A. cantonensis* infection in snails is an important component of epidemiological surveillance and the control of angiostrongyliasis.

**Methods:**

In this study, a new method for diagnosing *A. cantonensis* infection in gastropods was developed by recovering larvae from the buccal cavity of three snail species. The entire buccal cavity of a snail was extracted, and the tissue was pressed between two microscope slides to observe whether *A. cantonensis* larvae were present. Our new method was compared with traditional pathogenic detection methods of lung microscopy, tissue homogenization, and artificial digestion. We artificially infected 160 *P. canaliculata*, 160 *Cipangopaludina chinensis*, and 160 *Bellamya aeruginosa* snails with *A. cantonensis*. Then, the four different detection methods were used to diagnose infection in each snail species at 7, 14, 21, and 28 days post exposure.

**Results:**

We found no significant difference in the percentages of infected *P. canaliculata* snails using the four methods to detect *A. cantonensis* larvae. The radula pressing method had a mean detection rate of 80%, while the lung microscopy (81.3%), tissue homogenization (83.8%), and artificial digestion (85%) methods had slightly greater detection rates. Similarly, the percentages of infected *C. chinensis* snails that were detected using the radula pressing (80%), tissue homogenization (82.1%), and artificial digestion (83.8%) methods were not significantly different. Finally, the percentages of infected *B. aeruginosa* snails that were detected using the radula pressing (81.3%), tissue homogenization (81.9%), and artificial digestion (81.4%) methods were not significantly different. These results showed that the radula pressing method had a similar detection rate to traditional lung microscopy, tissue homogenization, or artificial digestion methods.

**Conclusions:**

This study demonstrates a new method for the qualitative screening of gastropods that act as intermediate hosts of *A. cantonensis* (and other *Angiostrongylus* species), provides technical support for the control of human angiostrongyliasis, and furthers research on *A. cantonensis*.

**Graphical Abstract:**

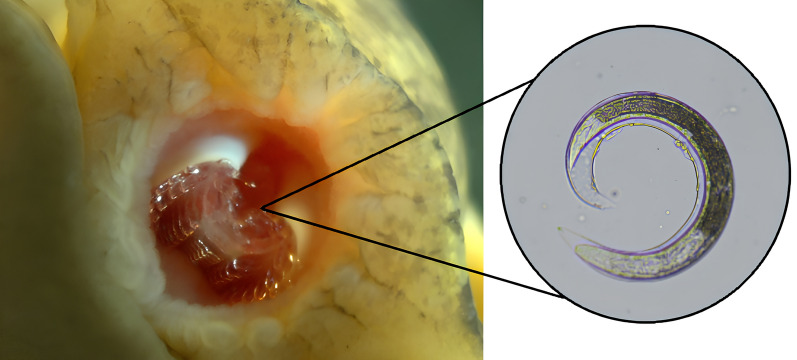

## Background

*Angiostrongylus cantonensis* (Chen, 1935) (Chromadorea: Angiostrongylidae) is a species of lungworm found in China and in many tropical regions around the world [1]. Since human infection by *A. cantonensis* was first reported in Taiwan in 1945 [2], the nematode has been recognized as an emerging zoonotic pathogen and has now caused thousands of cases of angiostrongyliasis worldwide [3]. Angiostrongyliasis is a zoonotic parasitic disease caused by the ingestion of the infective third-stage larvae of *Angiostrongylus* [4, 5]. The first reports of angiostrongyliasis occurred in Guangdong Province in 1933 [2]; the larvae of *A. cantonensis* were first discovered in rats in Guangzhou, and *A. cantonensis* was officially recognized as the cause of angiostrongyliasis by Chen in 1935 [1, 3].

In 2002, the World Health Organization (WHO) warned against *A. cantonensis* as an emerging food-borne parasitic disease; in 2004, it was listed as an emerging infectious disease in China [6]. In recent decades, major outbreaks have been reported in many endemic regions, especially in Mainland China [3]. Most cases of human angiostrongyliasis are due to the consumption of undercooked snails, fish, shrimp, vegetables, fruits, or unboiled water [7] contaminated with stage III *A. cantonensis* larvae. After infection, the nematode invades the central nervous system of the body and causes fever, headache, and neck stiffness, which can lead to death in severe cases [8].

The intermediate hosts of *A. cantonensis* are gastropods from 49 families, including 199 species that can be naturally (or artificially) infected with *A. cantonensis* larvae, of which 10 families and 33 species have been reported in China [9]. In recent years, snails such as *Bellamya* (Jousseaume, 1886), *Cipangopaludina* (Hannibal, 1912), and *Pomacea* (Perry, 1810) have been extensively farmed or taken from the wild in large quantities because they are an abundant, nutritious food source and are seen as a delicacy in most parts of China. Detecting *A. cantonensis* infection in snails is an important component of the epidemiological investigation of angiostrongyliasis outbreaks. Gastropods play an essential role in the life cycle and development of *A. cantonensis* larvae [10]. Therefore, the ongoing invasion and persistent spread of medically significant gastropod species such as *Pomacea canaliculata* heighten the risk of *A. cantonensis* transmission and contribute to an increase in angiostrongyliasis cases in China [7, 11], Thailand [12–14], India [15], France [16], Germany [17], and other regions.

The prevalence of *A. cantonensis* infection in new regions has created the need for rapid detection methods that can be used in common gastropod species to measure infection in endemic areas and prevent angiostrongyliasis outbreaks. Currently, the detection of *A. cantonensis* larvae in snails is typically carried out via lung microscopy, tissue homogenization, or artificial digestion. However, each of these methods has limitations; For example, the lung microscopy method is fast, but it can only be used for gastropod species that possess a pulmonary sac (i.e., pulmonates). Similarly, tissue homogenization and artificial digestion [18] are reliable methods that can be used for all gastropod species, but they are time-consuming and unsuitable for large-scale fieldwork. Alternatively, molecular detection techniques such as polymerase chain reaction (PCR) or loop-mediated isothermal amplification (LAMP) are sensitive methods that can be used for all gastropod species, but these techniques are expensive and need to be combined with traditional methods [19]. A fast, simple detection method is needed for all gastropod species.

In this study, we investigated whether *A. cantonensis* larvae could be detected by extracting the tissue of the buccal cavity from infected snails and compressing it between microscope slides. The buccal cavity was selected because it is the first place larvae enter during the infection process, and all stage I larvae must pass through the buccal cavity to continue their development. We then compared the new radula pressing method with the established lung microscopy, tissue homogenization, and artificial digestion methods to evaluate its effectiveness in detecting *A. cantonensis* infection in common intermediate gastropod hosts.

## Methods

### Isolating and culturing *A. cantonensis* larvae

We purchased 20 male Sprague Dawley (SD) rats, approximately 200–250 g in weight, from Kunming Nuoshun Biotechnology. A total of 447 commercially available *P. canaliculata* snails (imported from Ruili City, Dehong) were purchased from markets located in Taixing, Dali, Yunnan Province, in 2021. Five of these snails were infected with *A. cantonensis*, which was detected via the lung microscopy method. Stage III larvae were extracted by homogenizing the lung tissue and isolating the nematodes. The larvae were identified using molecular methods and fed to male SD rats at the Dali Institute of Schistosomiasis Prevention and Control. Then, stage I *A. cantonensis* larvae were cultured by collecting feces from the infected rats for 39 days [20, 21] after the rats had been exposed to stage III *A. cantonensis* larvae.

### Collecting and rearing wild snails in the laboratory

The wild *P. canaliculata* [22], *Cipangopaludina chinensis* [23], and *Bellamya aeruginosa* [24] snails used for the experiment were collected around Erhai Lake in Dali, Yunnan Province, in 2021. The snails were identified morphologically and were tested to determine whether *A. cantonensis* infection was common at Erhai Lake. Either lung microscopy (*P. canaliculata*) or tissue homogenization (*C. chinensis* and *B. aeruginosa*) was performed on 50% of the snails collected. Among the approximately 500 wild snails that were tested, zero *A. cantonensis* infection was detected in the populations at Erhai Lake. The collected snails were kept at room temperature with an aerator pump and a water filter to simulate the field environment [25]. In total, 160 snails from each species were selected. The selected snails were healthy and active, and had undamaged shells; they were fasted for 48 h before being exposed to laboratory-reared stage I larvae of *A. cantonensis*.

### Artificial infection of snails

#### Isolation and counting of stage I larvae of *A. cantonensis*

Stage I larvae were obtained from fresh rat feces that were dissolved in a Petri dish filled with dechlorinated water at 30 °C. When the feces had fully dissolved, the solution was filtered through a glass funnel equipped with a 200-µm mesh and a latex tube connected to a measuring cup. After 60 min, most of the stage I larvae had entered the dechlorinated water, and the feces and nylon silk were removed. The filtered solution and the extracted stage I larvae were observed under a dissection microscope. Further information can be found in Li et al. [26].

#### Infection of Erhai Lake snails

After the snails had been fasted for 48 h, they were individually placed into small beakers containing stage I larvae. The snails were exposed to stage I larvae for 24 h [20] at a ratio of 1 snail to approximately 200 nematodes. After 24 h, the snails were kept at room temperature and monitored for 28 days.

#### Distribution of larvae in infected snails

As a preliminary test, we exposed *P. canaliculata*, *B. aeruginosa*, and *C. chinensis* snails to stage I larvae of *A. cantonensis* and then used tissue homogenization to examine the organs and tissues of the three species 2 weeks later. We examined the hepatopancreas, buccal cavity, and lung sac (*P. canaliculata* only) to explore the distribution of *A. cantonensis* larvae in laboratory-infected snails.

### DNA extraction, PCR amplification, and identification of larvae

When an infected snail was found, DNA was extracted from a single larva using a blood/cell/tissue genomic DNA extraction kit (Tiangen, Shanghai, China). The identity of the extracted sample was confirmed by amplifying *ITS2* using the Ac-ITS2 (forward: 5′-ACG TCT GGT TCA GGG TTG TT-3′ and reverse: 5′-TTA GTT TCT TTT CCT CCG CT-3′) primer set. PCR was performed using 12.5 μl of 2 × Taq Master Mix, 8.5 μl of water, 1 μl of forward and reverse primers, and 2 µl of DNA template. The PCR cycling conditions were as follows: 94 °C for 3 min, followed by 35 cycles of denaturation at 94 °C for 1 min, annealing at 48 °C for 30 s, extension at 72 °C for 1 min, and final extension at 72 °C for 7 min. All PCR products were purified and sequenced in the forward direction by Sangon Bioengineering (Shanghai). The *ITS2* sequences then had their forward and reverse primers removed, and the quality scores for each of the nucleotide base were checked using FinchTV 1.4 (Geospiza, Inc.). The *ITS2* sequences were then used to confirm the species of the larvae using the US National Center for Biotechnology Information (NCBI) standard nucleotide Basic Local Alignment Search Tool (BLAST) search engine to find the closest matching reference sequences in the GenBank database.

### Detection methods

#### Grouping snails for detection

After exposing the snails to stage I larvae, we divided the snail species into four groups of 40 snails. Each group was tested post exposure at 7, 14, 21, and 28 days. In total, 160 snails were tested for each of the three species. This procedure was performed for each detection method, which resulted in 640 *P. canaliculata*, 480 *C. chinensis*, and 480 *B. aeruginosa* snails being tested.

#### Lung microscopy

**Fig. 1:**
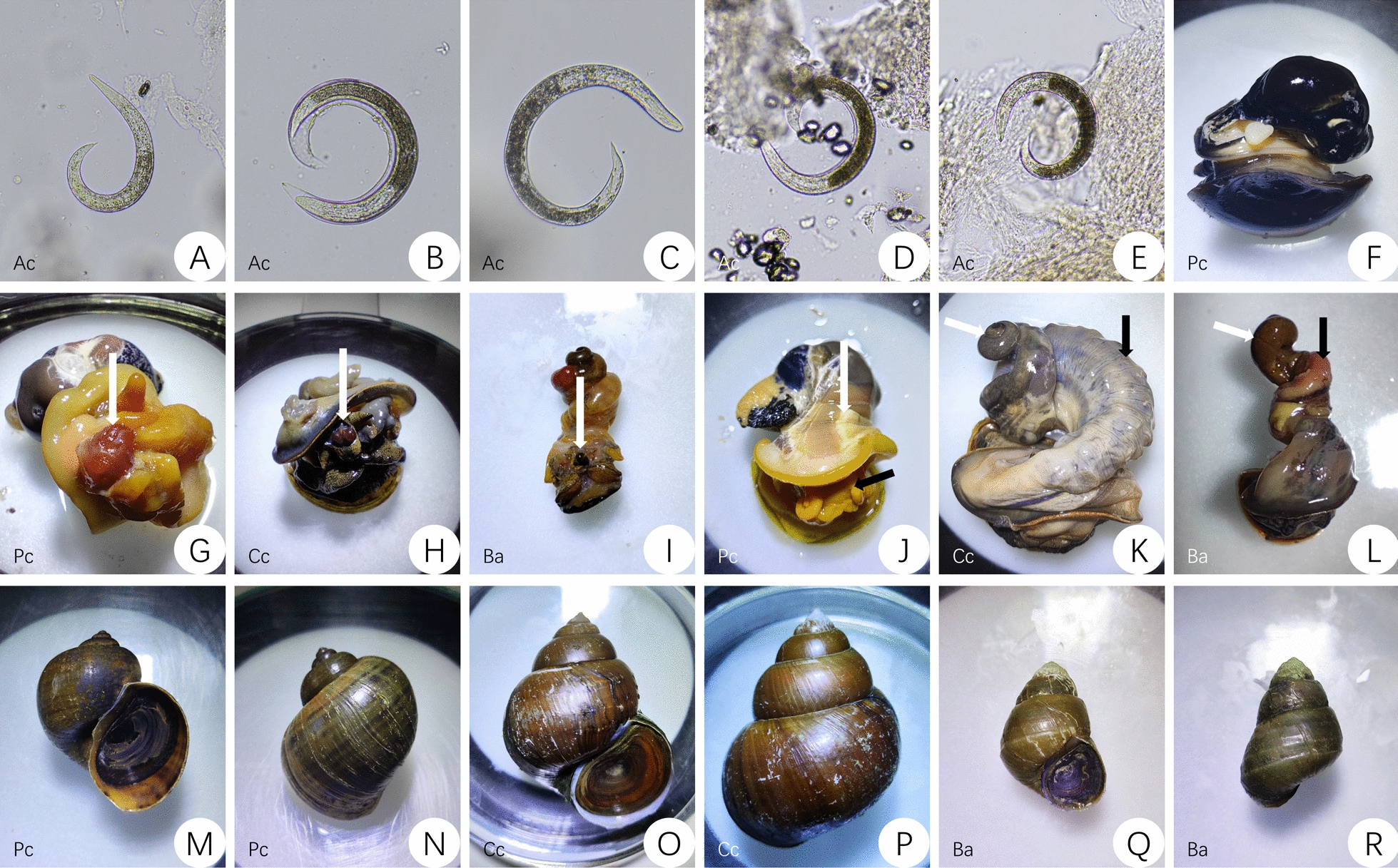
Morphology of stage I, II, and III larvae of *A. cantonensis* (Ac) and the anatomy of *P. canaliculata* (Pc), *C. chinensis* (Cc), and *B. aeruginosa* (Ba) snails. **A** Larvae of stage I *A. cantonensis*. **B** Larvae of stage II *A. cantonensis* and the secondary outer sheath. **C** Larvae of stage III *A. cantonensis*. **D** and **E** *A. cantonensis* larvae detected using the radula pressing method. **F** The anatomy of *P. canaliculata*; the white arrow indicates the heart. **G**, **H**, and **I** show the buccal cavity and radula knobs of dissected *P. canaliculata*, *C. chinensis*, and *B. aeruginosa* snails, respectively. **J** The frontal anatomy of *P. canaliculata*, with the white arrow showing the lung sac and the black arrow showing the head. **K** The frontal anatomy of *C. chinensis*, with the white arrow showing the hepatopancreas and the black arrow showing the gonads. **L** The frontal anatomy of *B. aeruginosa*, with the white arrow showing the hepatopancreas and the black arrow showing the gonads. **M** and **N** The front and back of *P. canaliculata*, respectively. **O** and **P** The front and back of *C. chinensis*, respectively. **Q** and **R** The front and back of *B. aeruginosa*, respectively

Lung microscopy is the preferred method for screening *A. cantonensis* infection in *Pomacea* and *Achatina* (Lamarck, 1799) snails, but it is applicable only to gastropod species that have a lung sac. This method was performed for *P. canaliculata* snails by removing the body of the snail from the shell with the operculum facing down. The lung sac was removed and opened using a scalpel and tweezers [27]. The lung sac was then laid flat under a dissection microscope to observe whether larval nodules occurred in the lung tissue [18, 28, 29]. An example of *A. cantonensis* found in lung tissue is shown in Fig. 1A–C.

#### Tissue homogenization

Tissue homogenization is used to screen for *A. cantonensis* infection in gastropod hosts that are too small for lung microscopy or do not have a pulmonary sac, or to extract *A. cantonensis* larvae from lung tissue. This method was performed by placing the lung sac (*P. canaliculata*) or the whole body (*C. chinensis* and *B. aeruginosa*) into a tissue grinder. The ground tissue was mixed with dechlorinated water and filtered through a 200-µm mesh sieve placed on a Petri dish. The mesh sieve was rinsed with saline, and the rinsed residue was placed under a dissection microscope to observe whether larvae were present. If found, larvae were extracted using a pipette and placed onto a slide under a light microscope for morphological identification [28, 30].

#### Artificial digestion

Artificial digestion is similar to tissue homogenization but uses a digestive solution to simulate the process of ingestion by a definitive host. This method was performed by cutting the lung sac (*P. canaliculata*) or the whole body (*C. chinensis* and *B. aeruginosa*) into small pieces and placing those pieces into a Falcon tube containing 25 ml of digestive solution (7 ml of hydrochloric acid and 5 g of pepsin per 1000 ml). The solution was heated to 37 °C for 1−2 h using a water bath. After digestion, the solution was filtered through a 200-µm mesh sieve. The mesh sieve was rinsed with saline, the rinsed residue was placed into a Petri dish, and a dissection microscope was used to observe whether larvae were present. If found, larvae were extracted using a pipette and placed onto a slide under a light microscope for morphological identification [31–33].

#### Radula pressing

Our radula pressing method is similar to lung microscopy, but instead of the lung sac, we use the peripheral tissues of the buccal cavity to determine whether *A. cantonensis* larvae are present in the radula and radula knobs. Our method is simple and fast (similar to lung microscopy), and it can be used on all gastropod species. The snail was first removed from the shell by removing the operculum and hepatopancreas. The head was sliced in half with a vertical incision down the middle of the snail’s mouth until the radula and radula knobs were exposed. The peripheral tissues of the buccal cavity containing the radula and radula knobs were removed, cut into thin slices, and placed between two microscope slides, which were pressed firmly together until the tissue became translucent. The slides were then observed under a light microscope. Stage I larvae were often elongated and active (Fig. 1A). Stage II larvae were slightly thicker and longer than stage I larvae and had an outer sheath (Fig. 1B). Stage III larvae were slightly thicker and longer than stage II larvae (Fig. 1C) and had the slowest activity [34].

### Observation indicators

The rates of detection of *A. cantonensis* in the three snail species were recorded for each detection method. The larvae at each developmental stage and the anatomical structures of each snail species were photographed (Fig. 1).

### Statistical analysis

Statistical analysis was performed using Microsoft Excel and SPSS 26 (IBM, Armonk, USA). To test whether there was a significant difference in the rate of detection between each method, a Pearson’s chi-square (*χ*^2^) test was performed in SPSS.

## Results

### The distribution of larvae in infected snails

When we examined the different organs and tissues of *P. canaliculata* snails exposed to *A. cantonensis* larvae, we found that most larvae were present in the lung sac (Fig. 1A–C) and buccal cavity (Fig. 1D–E), while the hepatopancreas contained only a small number of larvae. Similarly, in *C. chinensis* and *B. aeruginosa* snails, most larvae were found in the buccal cavity while only a small proportion of the larvae were found in the hepatopancreas.

### Confirming the identity of the larvae

When stage III larvae from infected snails were recovered, we found that their *ITS2* gene fragment (702 bp) was identical to that of adult *A. cantonensis* larvae obtained from the rats used to rear the parasite (NCBI accession no. PP796387). Similarly, our isogenic *ITS2* sequences (PP796387) showed the highest similarity (query coverage 98%, percentage identity 99.28%) to *Angiostrongylus cantonensis* (OR790457.1) when we used NCBI BLAST.

### Differences in detection sensitivity among the methods

In *P. canaliculata* snails, *A. cantonensis* larvae were detected at rates of 81.3%, 83.8%, 85%, and 80% by the lung microscopy, tissue homogenization, artificial digestion, and radula pressing methods, respectively. Differences in detection rates between the four methods were not significant (*χ*^2^ = 1.791, *df* = 3, *P* = 0.617), showing that these methods had similar sensitivities for detecting larvae in *P. canaliculata* snails (Tables 1 and 2).

**Table 1:** The total number of infected intermediate snails using different detection methods at different periods post exposure

Days post exposure	Number of positive *Pomacea canaliculata*				Number of positive *Bellamya aeruginosa*			Number of positive *Cipangopaludina chinensis*		
	Pressing method	Lung microscopy	Tissue homogenate	Artificial digestion	Pressing method	Tissue homogenate	Artificial digestion	Pressing method	Tissue homogenate	Artificial digestion
7	23	22	25	26	23	24	25	21	24	24
14	33	35	36	36	34	35	35	34	35	35
21	34	35	36	36	36	35	37	34	36	37
28	38	38	37	38	37	37	38	37	38	38
Total	128/160	130/160	134/160	136/160	130/160	132 /160	135/160	126 /160	133/160	134/160

In total, 40 snails of each species were tested on days 7, 14, 21, and 28 post exposure. In total, 160 snails of each species were tested for each of the detection methods. Pressing method = the radula pressing method; Tissue homogenate = tissue homogenization

**Table 2:** The percentage of *A. cantonensis*-infected *P. canaliculata* snails using different detection methods at different periods post exposure

Days post exposure	Detection rate (%)				*χ*^2^	*P*	*df*
	Lung microscopy	Tissue homogenate	Artificial digestion	Pressing method			
7	55	62.5	65	57.5	1.042	0.791	3
14	87.5	90	87.5	82.5	1.371	0.712	
21	87.5	90	90	85	0.657	0.883	
28	95	92.5	97.5	95	1.099	0.777	
Mean (%)	81.25	83.75	85	80	1.791	0.617	

In total, 40 snails were tested on days 7, 14, 21, and 28 post exposure. In total, 160 snails were tested for each of the detection methods. Tissue homogenate = tissue homogenization; Pressing method = the radula pressing method

The detection rates for the tissue homogenization, artificial digestion, and radula pressing methods for *B. aeruginosa* snails were 81.9%, 84.4%, and 81.3%, respectively. Differences in detection rates were not significant (*χ*^2^ = 0.606, *df* = 3, *P* = 0.739), showing that these methods had similar sensitivities for detecting larvae in *B. aeruginosa* snails (Tables 1 and 3).

**Table 3:** The percentage of *A. cantonensis*-infected *B. aeruginosa* snails using different detection methods at different periods post exposure

Days post exposure	Detection rate (%)			*χ*^2^	*P*	*df*
	Tissue homogenate	Artificial digestion	Pressing method			
7	60	62.5	57.5	0.208	0.901	2
14	87.5	87.5	85	0.144	0.93	
21	87.5	92.5	90	0.557	0.757	
28	92.5	95	92.5	0.28	0.869	
Mean (%)	81.875	84.375	81.25	0.606	0.739	

In total, 40 snails were tested on days 7, 14, 21, and 28 post exposure. In total, 160 snails were tested for each of the detection methods. Tissue homogenate = tissue homogenization; Pressing method = the radula pressing method

The detection rates for the tissue homogenization, artificial digestion, and radula pressing methods for *C. chinensis* snails were 83.1%, 83.8%, and 80%, respectively. Differences in detection rates were not significant (*χ*^2^ = 1.6, *df* = 3, *P* = 0.449), showing that these methods had similar sensitivities for detecting larvae in *C. chinensis* snails (Tables 1 and 4).

**Table 4:** The percentage of *A. cantonensis*-infected *C. chinensis* snails using different detection methods at different periods post exposure

Days post exposure	Detection rate (%)			*χ*^2^	*P*	*df*
	Tissue homogenate	Artificial digestion	Pressing method			
7	60	60	52.5	0.614	0.736	2
14	87.5	87.5	85	0.144	0.93	
21	90	92.5	90	1.19	0.552	
28	95	95	92.5	1.099	0.577	
Mean (%)	83.125	83.75	80	1.6	0.449	

In total, 40 snails were tested on days 7, 14, 21, and 28 post exposure. In total, 160 snails were tested for each of the detection methods. Tissue homogenate = tissue homogenization; Pressing method = the radula pressing method

### Advantages and disadvantages of each detection method

The lung microscopy method is fast and can be used to screen many snails at suspected outbreak sites, but it is only suitable for gastropod species with a pulmonary sac and requires advanced microscopy techniques [19] (Table 5). The tissue homogenization method has high sensitivity and can ensure that larvae are isolated from the host. However, the detection speed of this method is slow, and debris can obscure larvae. The artificial digestion method also has high sensitivity and results in less debris, making larvae easier to visualize. However, this method requires the longest detection time, and the addition of digestive solution reduces larval activity and can lead to larval death. Our technique, the radula pressing method, can be performed at a similar speed and workload relative to lung microscopy, but it is suitable for use in all gastropod species. However, radula pressing is slightly slower than lung microscopy and is not suitable for the isolation and collection of larvae (Table 5).

**Table 5:** Comparison of the advantages and disadvantages of each detection method to diagnose *Angiostrongylus cantonensis* infection in intermediate gastropod hosts

Method	Advantages	Disadvantages	Requirements for the method
Lung microscopy	Fast and simple to perform; Fast detection speed; Does not require much equipment to perform	Can only be used on gastropod species that have a pulmonary sac (e.g., pulmonates)	A gastropod with a pulmonary sac, body dissection, and a microscope
Tissue homogenization	The whole organ (or body) is used, reducing the chance of a false negative; Can be used on all gastropod species	Low clarity due to debris; Time consuming; Not as effective on larger specimens; Does not simulate the digestion process	Body dissection and a microscope
Artificial digestion	The whole organ (or body) is used, reducing the chance of a false negative, and it simulates the digestion process; Can be used on all gastropod species	Low clarity due to debris; Not as effective on larger specimens; The operation is more complex and time consuming than tissue homogenization; Can lead to the death and digestion of the parasite	Body dissection, artificial digestion, and a microscope
The radula pressing method	Simple to perform; Fast detection speed; Does not require much equipment to perform; Can be used on all gastropod species	Is not suitable for isolating and collecting worms from snail tissue	Body dissection and a microscope

## Discussion

*Angiostrongylus cantonensis* causes an emerging infectious disease in China. Many gastropod species are important intermediate hosts of *A. cantonensis* and play a key role in its transmission. The ability to detect *A. cantonensis* infection in gastropods is an important component of epidemiological investigation [35]. In this study, a new method of detecting *A. cantonensis* larvae in the peripheral tissues of the radula and the radula knobs was demonstrated to be simple, inexpensive, and labor-saving [18]. Furthermore, our results showed that the radula pressing method had a detection rate similar to that of lung microscopy, tissue homogenization, and artificial digestion. Although our method is easier and faster than lung microscopy, tissue homogenization, or artificial digestion, radula pressing cannot be used for separating larvae during the detection process (Table 5). However, this disadvantage can be solved by combining our technique with tissue homogenization or artificial digestion after confirming that a specimen is infected. A disadvantage to all microscopy methods is that *Angiostrongylus* larvae cannot be identified at the species level using morphology alone. Therefore, once larvae are separated, molecular detection methods should be used to confirm the presence of *Angiostrongylus* [36] and determine which species is present.

In this study, we found that the detection rates for the lung microscopy, tissue homogenization, artificial digestion, and radula pressing methods increased as the number of days post exposure increased. Stage I larvae are difficult to detect during the early days of infection owing to their small size. Therefore, the early stages of infection have a high false-negative rate of detection, and morphological detection should be used in conjunction with molecular detection methods. During this experiment, we found that pressing tissues (e.g., the hepatopancreas) other than the peripheral tissues of the radula knob led to poorer detection owing to their darker color or hardness, while the peripheral tissues of the radula knob were lighter in color and softer in texture, and had a high number of larvae present. Therefore, the peripheral tissues of the radula knob were chosen as the focus of observation.

### Improving the separation of stage I larvae from rat feces

Previous studies have outlined routine methods for separating stage I larvae. (1) Infected rat feces are added to dechlorinated water, allowed to fully dissolve, and allowed to settle for 4–6 h; (2) the sediment is collected after filtration, dechlorinated water is added, and the sample is thoroughly mixed; and (3) larvae are counted under a microscope [37]. However, when we used this method, we found that fecal suspensions appeared cloudy, with significant sedimentation, which compromised the accuracy of larval counting. Therefore, we propose optimizing the separation and counting of stage I larvae of *A. cantonensis* from laboratory-infected rat feces by: (1) preserving the protective mucus film on the exterior of fresh rat feces by not washing the feces, as its disruption may affect the quality of the fecal suspension and the subsequent accuracy of larval counting; and (2) allowing the feces stored at 4 °C to return to room temperature before the separation process to minimize the effect of temperature on nematode activity.

## Conclusions

In this study, we successfully established radula pressing as a new method for detecting *A. cantonensis* (and other *Angiostrongylus* species) infection in intermediate gastropod hosts, which provides technical support for the epidemiological investigation of angiostrongyliasis. Since this method has limitations in the isolation and identification of *A. cantonensis*, we suggest using it in combination with tissue homogenization and molecular methods, which will improve its accuracy.

## Data Availability

The datasets supporting the findings of this article are included within the paper. The *ITS2* sequence of the laboratory-reared *Angiostrongylus cantonensis* used in this study is available at GenBank (accession no. PP796387).
